# How Fast to Avoid Carbon Emissions: A Holistic View on the RES, Storage and Non-RES Replacement in Romania

**DOI:** 10.3390/ijerph20065115

**Published:** 2023-03-14

**Authors:** Adela Bâra, Simona-Vasilica Oprea, Niculae Oprea

**Affiliations:** 1 Department of Economic Informatics and Cybernetics, Bucharest University of Economic Studies, 010374 Bucharest, Romania; 2 Polytechnic University of Bucharest, 060042 Bucharest, Romania

**Keywords:** coal replacement, RES, non-RES, CO_2_ emissions, storage facilities

## Abstract

The European Union targets aim to replace the non-renewable energy sources (non-RES) of coal, oil and gas (COG) generation with RES and storage (RES-S). The replacement of COG-generating units will lead to a decrease in CO_2_ emissions and a better living environment. Starting from this desideratum, in this paper, we create several scenarios to replace COG in Romania with RES-S, reconsider future energy mixes and engage with a more creative planning in order to meet the clean energy transition path. The energy shortages, especially in European countries after the Russian invasion of Ukraine, led many governments (including the Romanian, Polish, etc.) to think more about short-term supply issues and less about medium- and long-term power system planning. However, the decision makers of the European power systems have to decide how fast to avoid firing coal, how fast to adopt RES and how fast to invest in flexibility sources, including storage stations to enable a higher integration of RES. Therefore, in this paper, a holistic view to envision the RES and non-RES contribution to the load coverage in Romania for a smooth transition to a low-carbon economy is provided. The results show that an initial mix of wind, photovoltaic (PV) and storage systems is preferable to substitute 600 MW of installed power in coal-based power plants. Furthermore, the case of Poland—the European country with over 70% coal in its generation portfolio—is also presented as it can serve as a good example.

## 1. Introduction

Phasing out coal would limit the global temperature increase to 1.5 degrees Celsius and help avoid climate change and harm to citizens’ health [1,2]. According to the International Monetary Fund, USD 29 trillion are required to substitute coal—“46% in Asia, 18% in Europe, 13% in North America, 13% in Australia and New Zealand, 8% in Africa, and 2% in Latin America and the Caribbean”. However, the gain is estimated to reach USD 78 trillion over the coming decades out of economic and health benefits. The International Monetary Fund also estimates that the private sector, end users, public–private partnerships and governments will provide the funds to replace coal with RES [3].

The potential of wind and solar systems to substitute coal was investigated for Texas [4] as in Texas, coal is still burned in large amounts and generates more CO_2_ than any other state in the USA although coal’s share fell to 20% in 2019, while RES grew to 22%. From the existing projects, using a mixed-integer optimization problem, the authors found that “72 of the 108 wind projects and 42 of the 262 solar projects in the queue” are sufficient to replace coal generation. They considered the characteristics of wind and photovoltaic (PV) systems that already operate in Texas that enable this displacement to be obtained. Other states were investigated from a similar perspective, such as Illinois [5]. The potential of PV systems to replace coal-fired power plants in Chinese cities was studied in [6] analyzing whether PV technology is economically feasible to replace coal-fired power plants in Chinese cities without subsidies. What degree of coal-fired power plants can be substituted by PV systems was first investigated. The authors took into account the investment feasibility and indices, such as net present value, payback period and internal rate of return and found that 65.99% of the cases could obtain a moderate to high financial return. 

The effects of a coal phase-out in European countries with regard to reaching the United Nations Sustainable Development targets were investigated in [7], especially the effects in the countries that massively export coal to Europe. Furthermore, the effects on RES technologies (such as wind, storage and PV systems) that are thought to replace coal-fired power plants were analyzed. The results obtained with an econometric model showed that a phase-out of coal-fired power plants would have numerous positive effects on biodiversity and water management, pollution and environmental issues and health at the societal level. 

The assessment of the air pollution emission reduction effect of the coal substitution in China was proposed in [8]. Furthermore, a gradual substitution and how to achieve the first steps of the carbon-neutrality 2060 target in China were investigated in [9,10]. A SWOT analysis is provided in [11]. The coal substitution perspective was also studied in other European countries [12,13], in the USA and in other Asian countries, such as Japan and South Korea [14,15]. Alternatives were to consider the nuclear option [16]. Many studies were focused on China [17,18], including the expansion of China’s solar energy and the impact of coal to gas replacement on air quality in Beijing. A study on the policy of replacing coal-fired with gas-fired boilers for central heating in Tianjin, China was proposed in [19]. The results showed that “grasping the appropriate proportion of clean heating and providing subsidies to heating enterprises are the keys for the government to implement the policy in a step-by-step manner to achieve good environmental and economic benefits”. The authors pointed out that “the proportion of heating by the gas-fired boiler is 34.7–35.7% and the subsidy price varies from 0.75 to 1.50 yuan/m3”, providing valuable insights for decision makers. Other studies also focused on coal to substitute natural gas based on a combined coal–steam gasification and one-step methanation [20] and to substitute natural gas production from different coal gasification processes based on modeling [21].

The energy transition from conventional coal-fired power plants to power systems based on RES has been initiated by imposing carbon taxes that gradually increased (https://www.investing.com/commodities/carbon-emissions-historical-data, accessed on 15 August 2022). Then, there was more pressure by a new geopolitical crisis that stared in February 2022, when the Russian invasion started in Ukraine that raised energy prices to unprecedented levels [22]. The price were 6–7 times higher in August 2022 on the European day-ahead markets (https://euenergy.live/, accessed on 15 February 2023). Furthermore, COVID-19 put pressure on electricity prices as immediately after lockdowns, the demand for commodities was much higher leading to a higher index price [23]. The war in Ukraine, in the Black Sea region, also stimulated the demand for commodities. Furthermore, the price of RES technology and even storage has gradually decreased, and new mines have been discovered in Africa that allow these technologies to evolve, including batteries for electric vehicles (EVs) [24]. PV systems became affordable, the European Union provided grants, and more and more energy communities [25] and prosumers have emerged [26,27,28]. However, the replacement of conventional cars with EVs at large scale will transfer the pollution burden from the transport sector to the energy sector. More energy will be required to cover the load to supply EVs; therefore, more RES will be necessary to benefit from a better living environment [29,30].

Poland, like Romania, is also very close to the war in Ukraine, and, moreover, coal plays a greater and critical role in Poland. In 2020, Poland announced the intention to gradually abandon the coal industry that covered 72.4% in electricity generation in 2021. The target is to close the mines by 2049. This target was reached after long negotiations with mining unions. It was more a psychological barrier that was overcome because the largest coal producer in Europe decided to end coal mining. The transition can be ahead of schedule as the experience with RES in Poland is also unique and interesting [31,32]. Poland focused more on testing various business models and experiments. The energy cluster concept was invented in 2016 to conceive new regulations and frameworks for successful operation of the clusters as energy or RES communities. It allows local entities, including municipalities, to create and test new business models for cluster operation. Energy Cluster in the Gliwice District, Friendly Energy, Baligród Renewable Energy Microcluster, Virtual Green Ochotnica Power Station Energy Cluster, etc. are several energy cooperatives that emerged from this concept [33]. Germany and Denmark have offered a classical approach, such as incentives and trading privileges to energy communities, whereas Poland, the Netherlands and the UK have provided support for new business models, experiments and innovation in terms of testing and regulating successful business cases in order to spread the development of the energy communities [34].

To come back to the coal critical share of coal in the power generation in Poland, the decision to end coal mining was accentuated by the negative effect on the total energy demand caused by the COVID-19 pandemic [35]. Furthermore, cheaper coal and renewable energy competition, unprofitable mines, aging coal-fired power plants and ever-increasing carbon certificate prices strengthen the position toward ending coal mining in Poland. Competition, aging and additional costs for CO_2_ certificates increase the generation costs, and therefore the market share of the coal industry in Poland has been slowly decreasing. The Polish government planned to invest in RES and nuclear power plants to substitute coal, and gas is likely to be fired during the transition period. Since more than 70% is covered by coal, the stability of the Polish power system is essential. The concern is also related to the costs of the transition that could be much higher than the current issues with coal [36,37]. Subsidies for PV system installations have been offered to consumers to make them more active and less affected by the transition. Apart from costs, two issues are still very sensitive, requiring responsible management [38]: (1) reconversion opportunities for miners [39] and other related jobs that can be applied to the new industries that emerge during the transition that require new workers and (2) environmental issues. According to the Centre for Public Opinion Research, 74% of Poles support the substitution of coal-fired power plants, regarding it as an energy source from the past. Furthermore, coal-fired power plants are harmful and have caused numerous deaths and premature deaths especially in Poland, but neighboring countries were also affected, and yet other tensions were provoked by the effects of coal.

Several studies regarding the replacement of COG were recently performed [40,41]. In [41], the replacement of coal-based power plants by photovoltaic (PV) systems in the Portuguese power system was envisioned. The results showed that coal-fired plants could be replaced by around 8000 MW of a PV system plus a small increase in the hydropumping capacity. In this scenario, CO_2_ emissions dropped by 56%. In [40], a substitution of coal power plants (of 600 MW) with RES considering the shift of the load and energy storage was also proposed. In their study, the hydrogen storage offered the best alternative to replace the coal-fired power plants in North Texas. The authors optimized the storage capacity with hydrogen considering the power substituted by wind and PV or solar, PV percentage and wind percentage.

As biodiversity is affected and the number of humans and the amount of consumerism are increasing, greenhouse gases (GHGs) massively impact human health. By deforestation, the extinction of species is threatening biodiversity and human civilization as numerous viruses emerge (such as Ebola, SARS-CoV-1, SARS-CoV-2, COVID-19, MERS, etc.) since the living chain is affected, disturbing the trophic levels. Although COG-based power plants are one of the main polluters, the transportation sector plays a more critical role, and special care should be taken to minimize the health risks [42,43]. Moreover, individual actions are essential in order to encourage proenvironmental measures, such as the usage of ecofriendly products [44], selective waste collection and e-waste recycling [45,46]. Climate change and its implications in Nigeria are studied in [47], emphasizing the major threats facing human health as usually developing countries suffer more from poverty and tend to cut trees to plant commercial crops (soya and palm trees for oil). Assisting developing countries in handling poverty and changing behavior from harsh exploitation to ecotourism and green-friendly initiatives is therefore essential. Due to intensive globalization, people are travelling often by plane, and the spread of diseases is very rapid, which can be deadly for millions of people. Special measures to limit airplane-boarding methods that reduce the risk of spreading viruses can be envisioned [48]. Issues threatening climate change are also analyzed in [49,50,51] where the authors point out the energy efficiency and GHG emission mapping of buildings and energy consumption policy and climate change effects in Algeria [52] and in Bangladesh [53].

In this paper, we aim to describe the Romanian case in terms of primary resources, total generation and load and understand the potential to substitute COG and offer a holistic view on RES, storage and non-RES replacement in order to avoid carbon emissions in the future. This analysis is mainly based on the operating characteristics of RES (PV and wind systems) that were constant in terms of installation in the last three and a half years allowing us to know their operation capabilities in weather conditions specific to Romania. The contribution of this paper consists in performing an analysis on the RES and storage potential in Romania to replace COG. We investigate the operation particularities of PV systems and wind at the power system level in the analyzed intervals and create reasonable assumptions to calculate the required new RES capacity and storage to replace COG. To the best of our knowledge, this investigation is novel, and the results provide interesting insights for policy makers in terms of costs and CO_2_ emissions.

This paper is structured in five sections. In the current section, the context and other related studies are depicted. Section 2 is dedicated to the input data analysis focusing on the most relevant and recent years 2019–2022 and using open-source data. In Section 3, considering the typical daily load curve in the most recent years, we depict several assumptions and formalize the overall relations among RES, storage and non-RES in order to create replacement scenarios that are described in Section 4. The assumptions consider the experience with RES operation in Romania and its characteristics (for wind and PV systems). Discussions and conclusion are presented in Section 5.

## 2. Input Data Analysis

**Figure 1:**
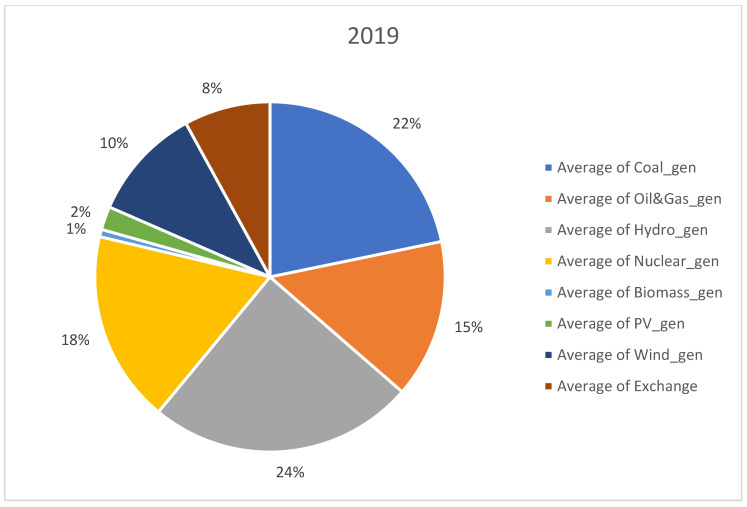
Distribution of generation sources in 2019.

**Figure 2:**
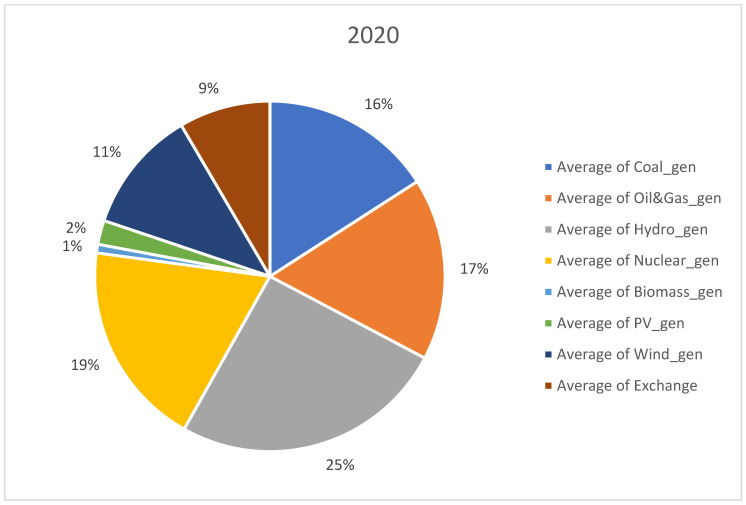
Distribution of generation sources in 2020.

**Figure 3:**
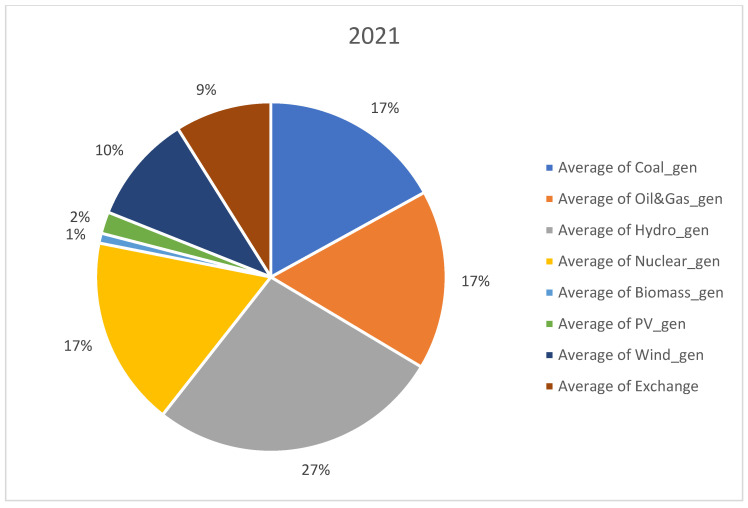
Distribution of generation sources in 2021.

**Figure 4:**
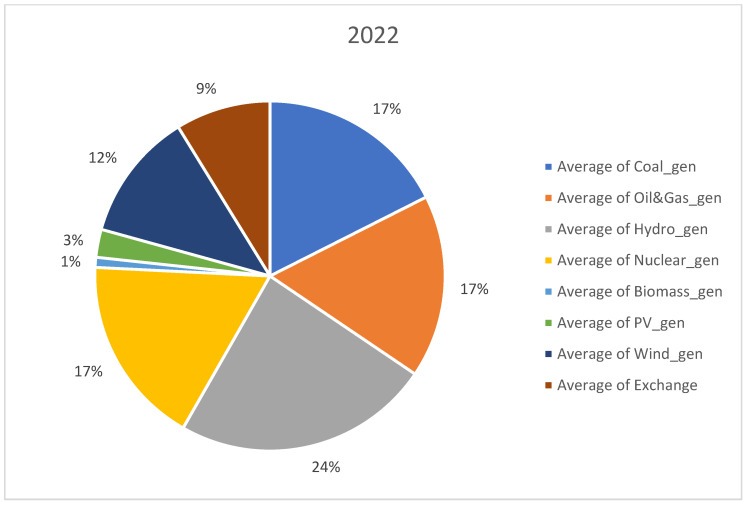
Distribution of generation sources in 2022.

For analysis, we considered open data sets recorded from 1 January 2019 to 31 August 2022. The data set was downloaded from Transelectrica website (https://www.transelectrica.ro/widget/web/tel/sen-grafic/-/SENGrafic_WAR_SENGraficportlet, accessed on 15 August 2022). It consists of hourly records of total consumption, total generation and its breakdown or generation by categories (coal, oil and gas, hydro, nuclear, wind, PV and biomass) plus the exchange or sold of the Romanian power system with the neighboring power systems. The selected above-mentioned interval represents the most recent three and a half years with hourly power system records. The interval consists of more than one year before the COVID-19 outbreak and almost 6 months after the Russian invasion in Ukraine. Furthermore, before 2019, the electricity markets were more stable, and there were not so many random events, such as the COVID-19 pandemic disease and conflict in Ukraine, that have had a significant impact on the energy sector. When collecting the data set, we did consider a longer past interval; it did not provide more insights regarding our approach that is a holistic view or an opinion on the RES, storage and non-RES replacement in Romania. A detailed distribution on generating resources is provided in Figure 1, Figure 2, Figure 3 and Figure 4 for each year from the 2019–2022 interval.

No significant change took place in the distribution of generation sources in the studied interval, expect RES generation slightly increased from 12% in 2019 to 15% in 2022. Furthermore, coal generation decreased from 22% to 17%.

**Figure 5:**
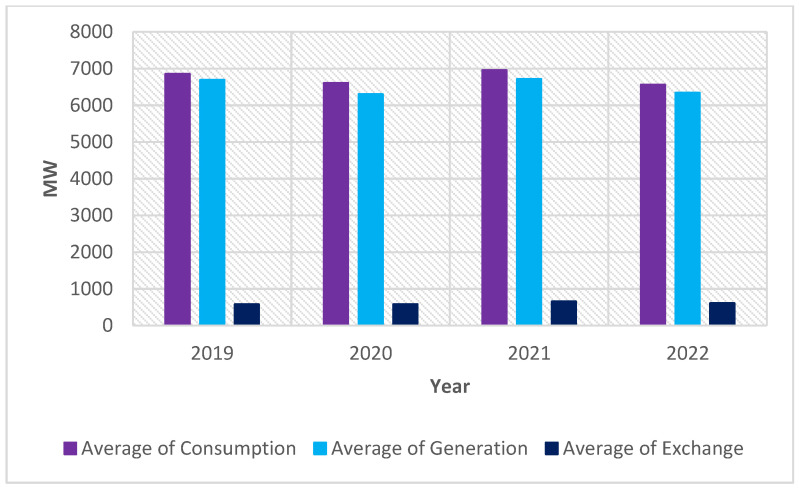
Average electricity consumption and generation between 2019 and 2022 in Romania.

In Romania, on average, consumption is slightly higher than generation. It totals up to 6500 MW in the first eight months of 2022, decreasing by 300–350 MW in comparison with 2019 and by 400 MW in comparison with the previous year as in Figure 5.

**Figure 6:**
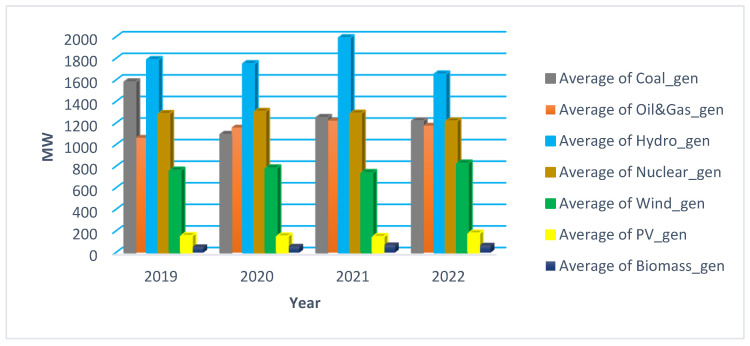
Average contribution to the hourly consumption from 2019 to 2022.

The generating units based on resource type distribution that covered the load year by year from 2019 to 2022 are depicted in Figure 6. Both non-RES (nuclear, coal, oil and gas) and RES (including hydro) contributed to supply the consumption (as in Figure 6).

**Figure 7:**
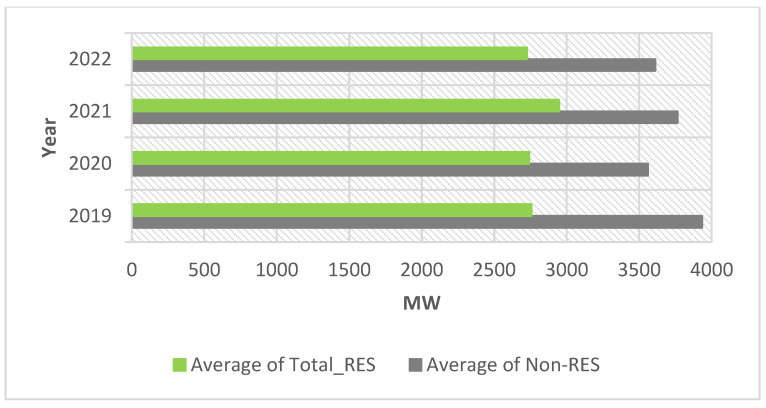
Total RES and non-RES from 2019 to 2022.

The contribution of non-RES is higher than the contribution of RES to the load coverage, but the contribution of non-RES decreased from 3934 MW in 2019 to 3611 MW in 2022 (as in Figure 7). The Pearson correlation coefficients were calculated for each year and are provided in Table 1, Table 2, Table 3 and Table 4. Between wind and solar there is an inverse weak correlation (marked in yellow) for the entire interval.

It is interesting to observe the evolution of the installed power in RES, especially in wind and PV. No significant power was installed in the analyzed interval as it can be remarked in the yellow cells of the above tables.

**Figure 8:**
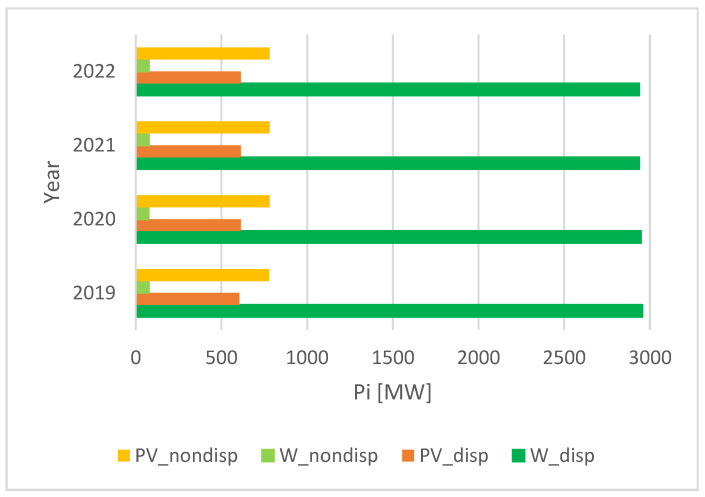
Installed power in wind and PV systems in Romania, 2019–2022.

The total power for wind totals up to 3000 MW, whereas the dispatchable PV-installed unit power is up to 612 MW (as in Figure 8). The non-dispatchable PV-installed unit power is even higher, around 780 MW. Therefore, no significant investment took place during the last 3–4 years in RES in Romania.

## 3. Materials and Methods

**Figure 9:**
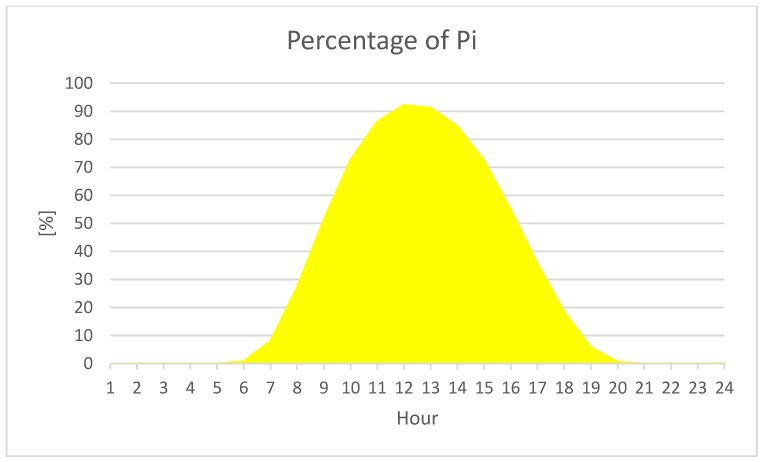
Average hourly operational output of the PV systems in Romania, in percentage.

In order to analyze the potential of replacement of COG in the Romanian power system, we analyzed the hourly curves during 2019–2022. For the entire interval, COG follows the load curve adjusting the output according to the peak and off-peak hours, whereas PV and wind do not follow the load curve. The wind generation is rather constant, but for the PV generation, the bell curve is displayed. Furthermore, we noticed that wind generation is about 30% of the installed power (27.63%), whereas PV generation at noon can reach 92% of the installed power—Pi (as in Figure 9).

**Figure 10:**
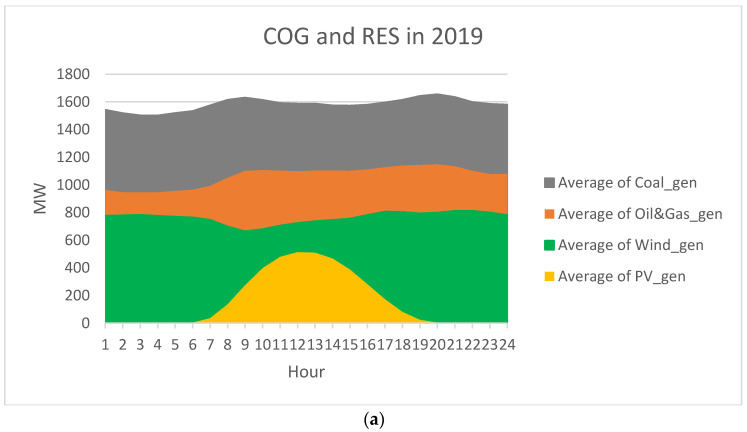

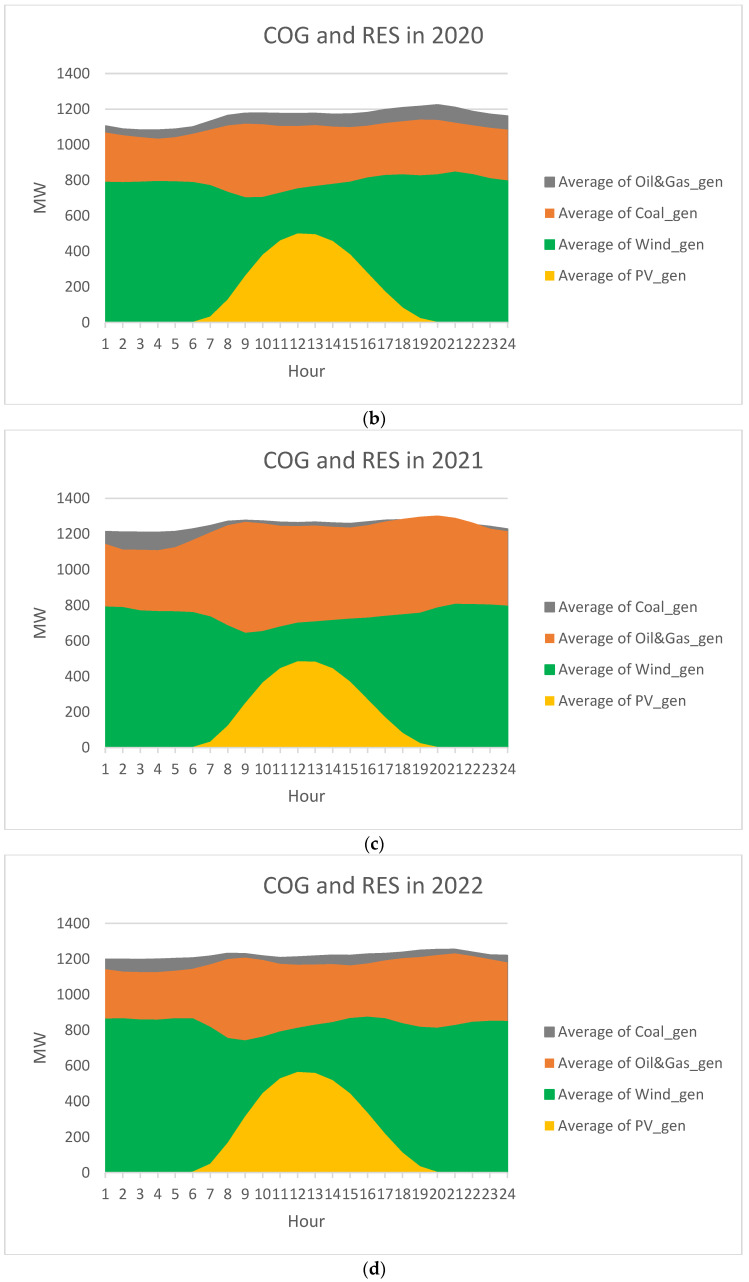
COG and RES in 2019–2022 (**a**–**d**).

However, the bell distribution is very different from the almost constant wind-generated power distribution. The PV systems, on the other hand, have zero output in the evening and at night; therefore, their profile is highly inflexible (as in Figure 10). If the PV systems could produce constantly, the hourly average would be around 179–180 MW; that is 29.34% of the installed power. From Figure 6, we find that the COG share and the distribution of the PV generation particularly complicate the replacement of COG. 

Another aspect can be noticed from Figure 10, namely that the coal coverage (the dark grey area) significantly decreased over the years. It was slightly compensated by the oil and gas coverage. Moreover, the total COG coverage decreased from 2646 MW in 2019 to 2396 MW in 2022. 

Therefore, the non-RES power that can be replaced in Romania (coal, oil and gas) is about 2400 MW. To replace this amount with wind power considering the current availability of wind energy ($\alpha^{w}$) (27.63%), the system would require around 8685 MW of power installed in wind power plants (WPPs)—scenario 1. However, the required power to replace COG could come almost equally from wind and PV systems plus batteries that can provide storage and supply energy in the evening and night hours. The availability of wind power $\alpha^{w}$ is the ratio between the average available wind power $\bar{P_{d}^{w}}$ and installed power $P_{i}^{w}$ multiplied by 100, whereas the newly required installed power in WPPs $P_{i}^{nw}$ is the ratio between the COG power $P_{COG}$ that has to be replaced and the availability of wind $\alpha^{w}$ multiplied by 100.

(1) $$\alpha^{w}=\frac{\bar{P_{d}^{w}}}{P_{i}^{w}}\times 100$$

(2) $$P_{i}^{nw}=\frac{P_{COG}}{\alpha^{w}}\times 100$$

In the second scenario, if we consider replacing $P_{COG}$ 50% with $P_{i}^{nw}$ and 50% with $P_{i}^{npv}$, $P_{i}^{nw}=\frac{P_{i}^{nw}}{2}$, and the $P_{i}^{npv}$ is calculated as follows:

(3) $$\alpha^{pv}=\frac{\bar{P_{d}^{pv}}}{P_{i}^{pv}}\times 100$$

(4) $$P_{i}^{npv}=\frac{P_{COG}}{\alpha^{pv}}\times 100$$

where $\alpha^{pv}$—availability of PV;

$\bar{P_{d}^{pv}}$—average available PV power; 

$P_{i}^{pv}$—installed power;

$P_{i}^{npv}$—newly required installed power in PV; 

$P_{COG}$—COG power to be replaced.

The bell power curve of the PV systems suggests that a coefficient of generation $\beta_{h}^{pv}$ can be hourly calculated. It is the ratio between the average hourly available power generated by PV systems $\bar{P_{d,h}^{pv}}$ and installed power $P_{i}^{pv}$ multiplied by 100.

(5) $$\beta_{h}^{pv}=\frac{\bar{P_{d,h}^{pv}}}{P_{i}^{pv}}\times 100$$

(6) $$P_{COG}={\alpha^{w}\times P}_{i}^{nw}+\beta_{h}^{pv}\times \frac{P_{i}^{npv}}{100}+P_{h}^{bat}$$

(7) $$P_{h}^{bat}={\alpha^{w}\times P}_{i}^{nw}-\beta_{h}^{pv}\times \frac{P_{i}^{npv}}{100}$$

Therefore, the total power ($P_{COG}$) that is to be replaced by RES-S is the sum of the power generated by WPPs ${\alpha^{w}\times P}_{i}^{nw}$, power generated by PV systems $\beta_{h}^{pv}\times \frac{P_{i}^{npv}}{100}$, considering $\beta_{h}^{pv}$ as in Equation (5) and the storage ($P_{h}^{bat}$) that can act as a generator at night when the batteries are discharged and as a consumer during the day when the batteries are charged.

## 4. Results

In order to replace 2400 MW with RES or RES-S, the required power could be provided by WPPs—scenario 1 or come almost equally from wind and PV systems plus storage facilities (as in Table 5)—scenario 2. In scenario 1, considering the assumptions from Section 3, the required installed power in WPPs to replace 2400 MW of COG is around 8685 MW.

**Figure 11:**
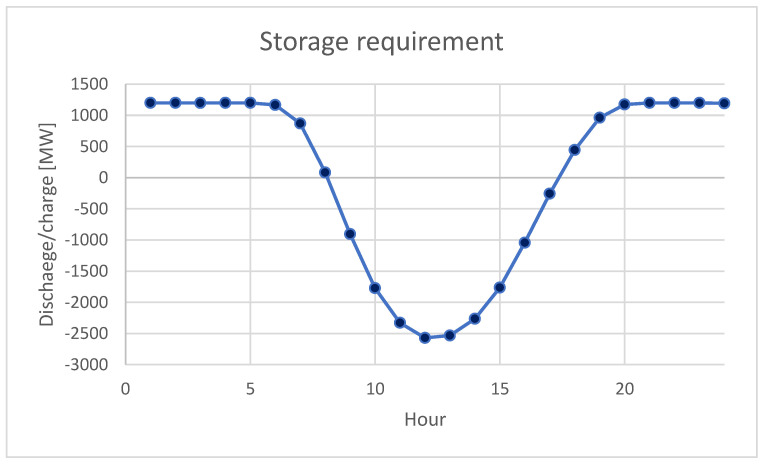
Storage capacity in scenario 2 in the case of 2400 MW replacement of COG.

In the second scenario, if the installed power in WPPs would be 4342.5 MW, the generated power from WPPs can be around 1200 MW; therefore, the storage requirement is depicted in Figure 11. The hourly storage requirements for night hours would be 1200 MW that together with 1200 from WPPs could lead to the replacement of 2400 MW generated now by COG. However, the required capacity of the battery systems is very high and very costly in this scenario. Thus, the total required capacity to replace 2400 MW of COG is above 15,000 MW.

Therefore, the target should be reduced; for instance, instead of 2400 MW to be replaced, we may consider a more reliable capacity, such as 600 MW as in [40]. Therefore, initially, we may target a substitution of 600 MW generated by coal-fired power plants with wind, PV and storage. However, gas-combined cycle units for 1 MWh produce 41.19% of the greenhouse gas emissions compared to coal-fired generating units. Therefore, it is preferable to replace coal first. In this case, in the first scenario, around 2200 MW of newly installed power in WPPs will be required or, in the second scenario, around 1100 MW in WPPs, 1022 MW in PV and 3900 MW in storage facilities (as in Table 6). 

The total cost with storage if the storage is based on batteries will be around EUR 1.9 billion, considering that the cost is EUR 500,000/1 MW.

In the first scenario, when around 2400 MW is replaced, on average 1.69 mil. tCO_2_ is avoided, whereas in the second scenario, 1.09 mil tCO_2_ is avoided. For the calculation, we considered that gas power plants produce 407.32 tCO_2_/MWh and coal-fired power plants produce 988.83 tCO_2_/MWh.

In addition, in this substitution scenario, the coal industry will not be affected immediately, and the transition is therefore milder. Some studies show that PV systems produce greater technoeconomic resource suitability than wind for replacing coal mining jobs [54]; therefore, a combination of wind and PV systems could be better. The effects of decarbonization on the energy system and related employment effects in South Africa are discussed at length in [55].

Even in the second scenario, considering a substitution of 600 MW, the costs are very high. However, other storage assets can also be considered, such as flexibility of the consumers/energy communities and projects of small hydropower plants with a storage capacity.

## 5. Conclusions

In this paper, we investigated a data set that consists of the total consumption, generation and its breakdown, recorded between 1 January 2019 and 31 August 2022 to understand the potential of RES and storage facilities to replace or substitute non-RES (coal, oil and gas) in the Romanian power system. Not only wind and PV systems can replace non-RES, but we chose to consider these sources as most investors who sent numerous connection requests to the public grid focused on wind, PV systems and even storage. Therefore, storage facilities are also gaining more interest, and the first storage units (Megalodon 7 MW) were installed, and there are new requests to connect to the grid in Romania, totaling significant individual power capacity.

The Polish experience can be very useful for Romania as Poland is the country with the most coal in its generation portfolio. The engagement of the Polish government to end coal mining by 2049 has raised many challenges, and probably the most important one is related to the energy stability in Poland. The conflict in the Black Sea region affects the decision and efforts to replace coal faster. Electricity markets are more volatile, and efforts are focused now to secure the internal supply during winters. Therefore, the transition to a clean energy environment is also influenced by random or less-expected events, such as the COVID-19 pandemic disease and the Russian invasion in Ukraine.

Poland is offering the growth framework for energy communities, encouraging the development of energy clusters with new regulations and business models in which value sharing is very important as it ensures the growth and the stability of energy communities. Similar steps have to be followed in Romania to form consumer coalitions and encourage communities to develop, making their members less affected by energy poverty and geopolitical instability.

By simulation, we found that to replace the entire COG (around 2400 MW), the effort would be very high. The storage capacity is large and costly in this scenario. In addition, tensions would appear because the mines were closed and unemployment would increase. A milder step of replacing 600 MW of coal-fired power plants with WPPs, PV systems and storage facilities is recommended. In this case, the newly installed power in WPPs should be around 1100 MW, in PV systems 1022 MW and in storage 3900 MW. The battery storage investment is estimated at EUR 1.9 billion. The cost compensations for coal companies that are shut down and for affected workers were not considered. However, the mix among wind, storage and PV systems is better than the wind-only scenario as it may also integrate more ex-coal workers.

Roughly 1.09 to 1.69 mil. t of CO_2_ can be avoided when replacing COG. However, the storage or alternatives could be provided not only by Li-ion batteries but load flexibility, storage with hydrogen, pumped storage hydropower plants and even new nuclear units can be a better solution than COG.

Climate change causes serious health problems, lung cancer and death that affect the entire human civilization as the local problems are not local anymore. Coal exploitation affects, for instance, air quality and drinking water quality in the local areas, but it can also extend to the neighboring countries, and more tensions appear between neighbors (as happened between Czech Republic and Poland). In addition, by destroying secular forests in countries where poverty is high, biodiversity is in great danger, and this affects large populations as we well know happened with the COVID-19 pandemic. The high number of flights between cities and countries as part of the transportation sector—the biggest polluter—also transport viruses and allow their spread very quickly. As we experienced with COVID-19, in just a couple of weeks, the entire planet was affected. However, COVID-19 was a mild virus in terms of mortality compared with other viruses that fortunately are not air-transmissible. Thus, in the future, climate change threatens humanity with more health problems and epidemies. 

Coal-fired power plants will be ultimately replaced even if the geopolitical problems in the Black Sea area may postpone this process, but what remains a concern is transportation problems that could be transferred to the energy sector as the load will increase significantly with the gradual increase in the number of EVs. Therefore, not only does coal substitution require more RES, but as the load at the low-voltage level increases and has to be supplied by additional sources, more RES are envisioned to play an important role. Due to their volatility, storage facilities must support RES, and these storage facilities must be designed at a transmission level where large hydropower plants (with storage lakes) usually operate, at a distribution or a medium-voltage level (such as Megalodon 7 MW connected to a medium-voltage level) and at the low-voltage level as flexibility offered by prosumers.

The replacement of coal is a complex process. That is probably why scientists have been concerned and have published many studies regarding this topic. There are numerous aspects that influence coal substitution: political will, huge costs, volatility of the electricity markets, unions and population pressures, consumption and load fluctuation and random events or the so-called black swans.

Regarding consumption, we expect that the residential consumption in Romania will have a slight decrease in the next few years due to energy efficiency measures (housing insulation), fewer energy intensive appliances, more Internet of Things (IoT) and distributed generation, but then it may increase as EV penetration gradually advances. Therefore, when consumption decreases, it will cost less to replace COG, whereas when consumption increases, more RES should be available to replace the harmful coal.

One limitation of this study is related to the fact that seasonality issues were not considered. In addition, the costs of closing mines and reconversion were not considered.

Our study focuses on the potential of RES and storage, and our aim in this paper is to provide a holistic view on this topic, probably one of the first in this area. The seasonality aspects and consumption variation must be studied thoroughly, and we are considering extending our future studies in this direction.

## Figures and Tables

**Table 1:** Correlation coefficients between consumption and generation sources in 2019.

2019	Consumption	Generation	Coal_gen	Oil and Gas_gen	Hydro_gen	Nuclear_gen	Wind_gen	PV_gen	Biomass_gen	Exchange
Consumption	1									
Generation	0.754999	1								
Coal_gen	0.52263	0.354775	1							
Oil and Gas_gen	0.567045	0.375932	0.425303	1						
Hydro_gen	0.182936	0.386768	−0.02498	−0.43178	1					
Nuclear_gen	0.174067	0.108548	0.205406	0.432251	−0.53019	1				
Wind_gen	0.118986	0.455295	−0.20786	0.084702	−0.24133	0.114054	1			
PV_gen	0.17712	0.180904	−0.06517	−0.16565	0.169873	−0.11078	−0.11567	1		
Biomass_gen	0.449508	0.239664	0.411848	0.698678	−0.52954	0.551382	0.171153	−0.09841	1	
Exchange	0.165864	−0.02581	−0.04925	0.078088	−0.05825	0.008436	−0.01483	0.02241	0.026157	1

**Table 2:** Correlation coefficients between consumption and generation sources in 2020.

2020	Consumption	Generation	Coal_gen	Oil and Gas_gen	Hydro_gen	Nuclear_gen	Wind_gen	PV_gen	Biomass_gen	Exchange
Consumption	1									
Generation	0.825083	1								
Coal_gen	0.583784	0.549967	1							
Oil and Gas_gen	0.66631	0.65791	0.697599	1						
Hydro_gen	0.463647	0.356033	0.137276	0.096338	1					
Nuclear_gen	0.085489	0.056769	−0.09498	0.088235	−0.48591	1				
Wind_gen	0.041043	0.436442	−0.14321	−0.04993	−0.34735	0.189985	1			
PV_gen	0.12561	0.087586	−0.12801	−0.17298	0.143236	−0.16636	−0.08603	1		
Biomass_gen	0.471238	0.40893	0.295541	0.460462	−0.15052	0.553423	0.16535	−0.22169	1	
Exchange	0.32365	0.091777	0.125158	0.098427	0.130642	0.112006	−0.14479	0.010451	0.180369	1

**Table 3:** Correlation coefficients between consumption and generation sources in 2021.

2021	Consumption	Generation	Coal_gen	Oil and Gas_gen	Hydro_gen	Nuclear_gen	Wind_gen	PV_gen	Biomass_gen	Exchange
Consumption	1									
Generation	0.719558	1								
Coal_gen	0.436387	0.291136	1							
Oil and Gas_gen	0.58185	0.409437	0.326192	1						
Hydro_gen	0.369665	0.561788	0.051653	−0.14258	1					
Nuclear_gen	0.110228	−0.01885	0.07934	0.275449	−0.4583	1				
Wind_gen	0.122566	0.550172	−0.14308	0.069502	−0.06679	−0.04133	1			
PV_gen	0.201324	0.158353	0.023534	−0.17176	0.187405	−0.10348	−0.16831	1		
Biomass_gen	0.317228	0.357262	−0.01	0.193739	0.286445	−0.01796	0.132848	−0.03331	1	
Exchange	0.215344	−0.0135	−0.00788	0.132373	−0.02943	−0.01787	−0.05167	−0.00961	−0.01011	1

**Table 4:** Correlation coefficients between consumption and generation sources in 2022.

2022	Consumption	Generation	Coal_gen	Oil and Gas_gen	Hydro_gen	Nuclear_gen	Wind_gen	PV_gen	Biomass_gen	Exchange
Consumption	1									
Generation	0.721791	1								
Coal_gen	0.263307	0.117945	1							
Oil and Gas_gen	0.42723	0.374905	0.082189	1						
Hydro_gen	0.417734	0.368431	0.279286	0.028125	1					
Nuclear_gen	0.281341	0.336698	−0.11292	0.095203	−0.31325	1				
Wind_gen	0.192882	0.648797	−0.2811	−0.07477	−0.17647	0.259488	1			
PV_gen	0.230318	0.180934	0.027002	−0.10864	0.17804	−0.14369	−0.10035	1		
Biomass_gen	0.429314	0.362305	−0.01691	0.182836	0.101654	0.233664	0.24608	−0.1063	1	
Exchange	0.279388	−0.0403	0.018273	−0.01893	0.032269	0.061683	−0.08281	−0.03774	0.151705	1

**Table 5:** The 2400 MW replacement case.

2400 MW Replacement Case	Pi Wind [MW]	Pi PV [MW]	Storage [MW]
Scenario1	8685	0	0
Scenario2	4342.5	4089.3	15,486

**Table 6:** The 600 MW replacement case.

600 MW Replacement Case	Pi Wind [MW]	Pi PV [MW]	Storage [MW]
Scenario1	2171.25	0	0
Scenario2	1085.62	1022.32	3871.50

## Data Availability

The data are open and available here: https://www.transelectrica.ro/widget/web/tel/sen-grafic/-/SENGrafic_WAR_SENGraficportlet.
